# Supplementary material for: Cell type-specific calcium imaging of central sensitization in mouse dorsal horn
Source: Nat Commun. 2022 Sep 3;13:5199. doi: 10.1038/s41467-022-32608-2 (PMC9440908; doi:10.1038/s41467-022-32608-2)
Supplement: Supplementary file 1 — Supplementary Information [file 41467_2022_32608_MOESM1_ESM.pdf]

Supplementary Information

Cell type-specific calcium imaging of central sensitization in mouse dorsal horn

Charles Warwick[1], Joseph Salsovic[1], Junichi Hachisuka[1,3], Kelly M. Smith[1], Tayler D. Sheahan[1], Haichao Chen[1,4], James Ibinson[2], H. Richard Koerber[1]*, and Sarah E. Ross[1,2]*

[1]Department of Neurobiology and the Pittsburgh Center for Pain Research, University of Pittsburgh, Pittsburgh, PA, USA

[2]Department of Anesthesiology, University of Pittsburgh, Pittsburgh, PA, USA

[3]Current address: Spinal Cord Group, Institute of Neuroscience and Psychology, University of Glasgow, Glasgow, United Kingdom

[4]Current address: School of Medicine, Tsinghua University, China

Author Emails
Charles Warwick: warwickc@pitt.edu
H. Richard Koerber: rkoerber@pitt.edu
Sarah E. Ross: saross@pitt.edu

This file includes:

Supplementary Figures 1–10

Supplementary Tables 1 & 2

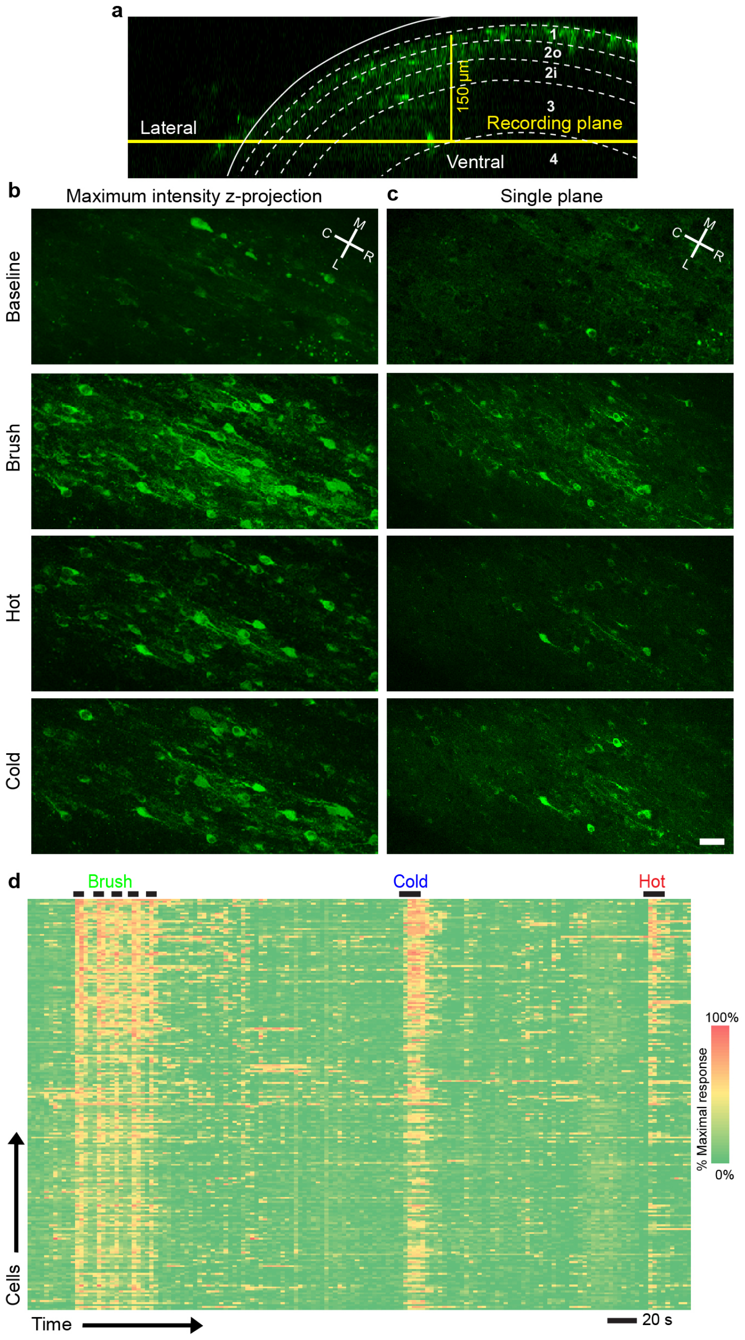

Supplementary Fig. 1 | Imaging cells beyond Lamina II provides similar signal to noise

a, GCaMP6s expression in *Vglut2-Cre* positive excitatory neurons in an orthogonal view showing a reconstructed XYZ image (*i.e.* a transverse slice) taken post imaging showing the recording position relative to the surface of the dorsal horn. **b**, Maximum intensity Z-projection of 3 image planes separated by 10 µm during brush, hot saline, or cold saline application. **c**, Individual imaging plane highlighted in **a** (scale bar, 30 µm). **d**, Heat map of Ca^{2+} responses in deeper lamina cell to brush, cold saline, or hot saline applied to the skin. N=2 animals for all graphs.

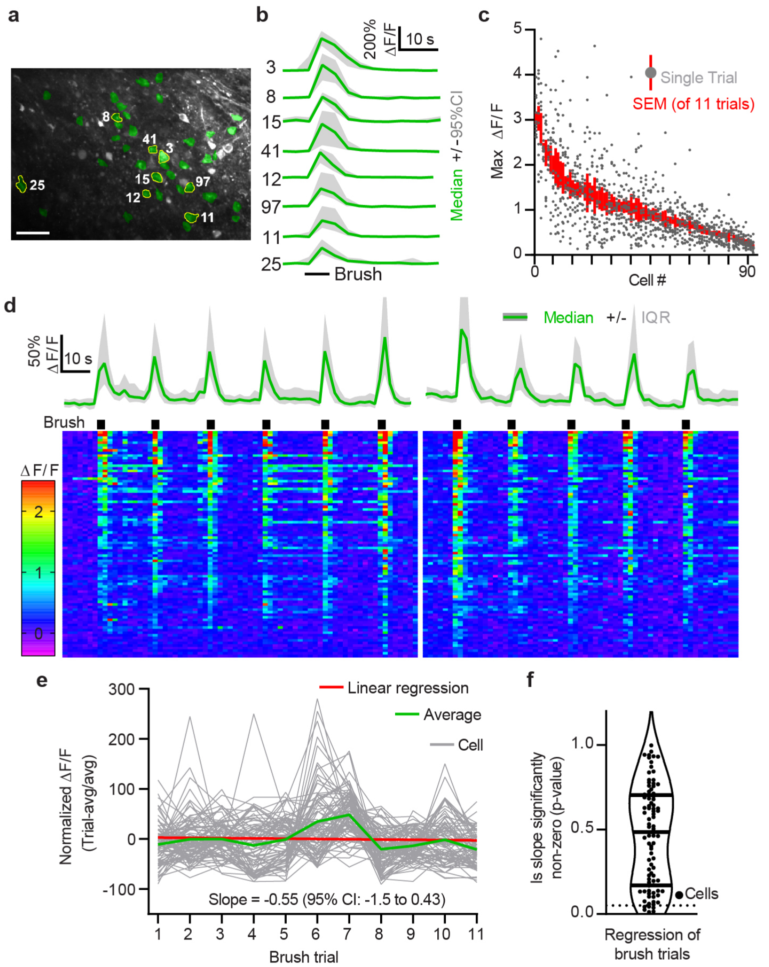

Supplementary Fig. 2 | Repeated cutaneous stimulation does not produce rundown of response

a, Superficial (<50 µm) GCaMP6s expression in *Vglut2-Cre* positive excitatory neurons. Brush responsive cells are highlighted in green (scale bar, 50 µm). N=11 animals. **b**, ΔF/F Ca^{2+} traces from the circled cells in **a**. Median (green) +/- 95% CI (grey) showing the consistency within 11 brush trials. **c**, ΔF/F Ca^{2+} amplitudes in response to brush in one example field of view. **d**, Top, ΔF/F Ca^{2+} traces median (green) +/- interquartile range (grey) of brush responsive cells. The second set of brush stimulations were applied after a one-hour break, which is indicated by the break in the axis. Bottom, heatmap of ΔF/F Ca^{2+} traces. **e**, Linear regression analysis of brush responses shows no correlation between the brush trial and the normalized response across the average of all cells. **f**, *P*-values of regression analysis to test whether the slope of each individual cell's brush responses are non-zero (The *P*-value is calculated from an F-test). As our hypothesis is that there is no significance and our Null Hypothesis is that there is a significant difference we did not apply multiple corrections in order to reduce the rate of false negatives.

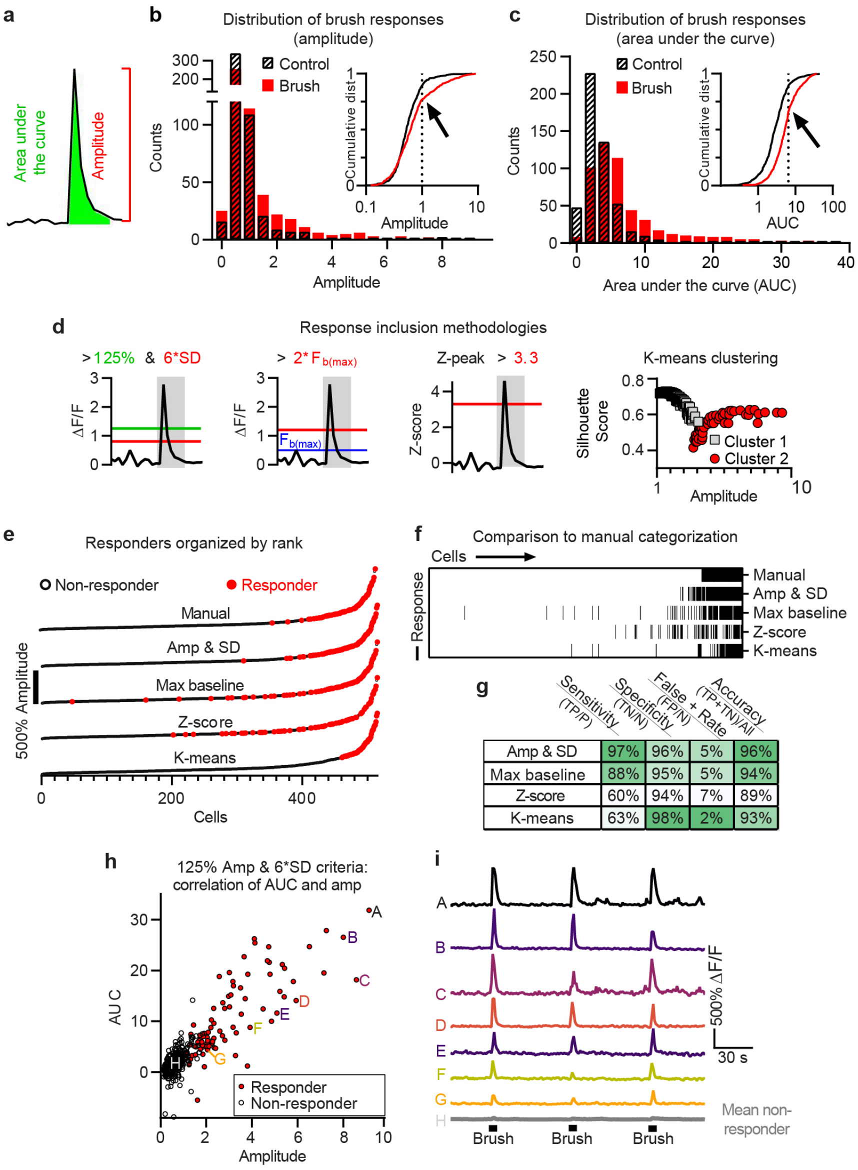

Supplementary Fig. 3 | Determination of threshold of response

a, Schematic of quantification metrics for Ca^{2+} signals. **b**,**c** Histogram of amplitude and AUC values from brush stimulation or an equivalent period of time immediately prior to stimulation. Inset: cumulative distribution of each value showing the inflection point at which two populations are readily distinguishable. **d**, Schematic of other published methods for determination of a ‘responsive’ neuron. **e**, Responses to brush organized by ranked amplitude response. Each line is the same set of cells determined as a responder (red) or non-responder (black) by the indicated method. **f**, Responses to brush ordered by manual categorization and then by amplitude. Black ticks indicate a responsive cell according to the indicated method. **g**, Comparison of different methods relative to manual review. True positive (TP), positive (P), true negative (TN), false positive (FP), negative (N). In general, we found that using a two-factor determination (*i.e.* Amplitude and SD) provided the best balance of the available methods. Methods like K-means had an exceptional specificity but very low sensitivity which could be preferable in some scenarios. Using the maximum fluorescence at baseline and also provided

very good overall accuracy at the expense of a noticeably smaller sensitivity. This is due to the high penalty incurred by having a small amount of spontaneous activity at baseline, which is generally not seen in DRG where the method was originally utilized [1]. **h**, Scatterplot of amplitude vs AUC with 125% & 6 SD criteria overlaid in red. **i**, Individual ΔF/F Ca^{2+} traces for labeled cells in **h**.

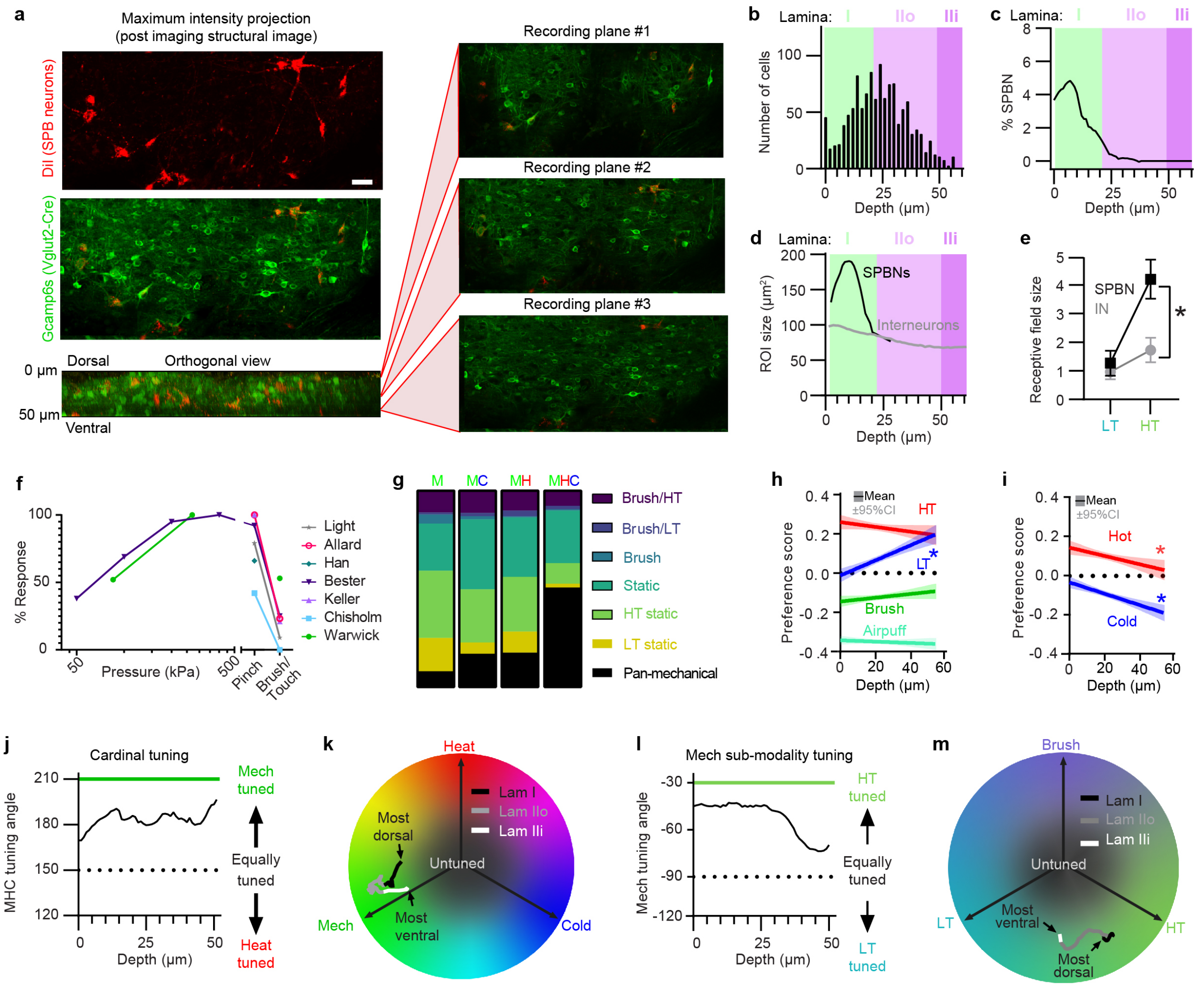

Supplementary Fig. 4 | SDH DV distributions affect sensory tuning

a, Example images of a typical field of view within the SDH showing GCaMP6s expression in *Vglut2-Cre* positive excitatory interneurons (IN, green only) and spinoparabrachial neurons (SPBN, red and green). Maximum intensity projection images (left) are shown of the 3 recording planes (right). Images are taken at the end of each experiment in the presence of a high potassium solution to visualize viable cell bodies (scale bar, 40 µm). N=11 animals. **b**, Histogram of sampled neurons location relative to the surface of the grey matter. **c**, % of cells at each depth which were SPB neurons. **d**, Average region of interest (ROI) size of SPBNs and INs at each recorded depth. **e**, Receptive field sizes for LT and HT stimuli in SPBNs and INs. SPBNs had a significantly larger receptive field sizes for HT stimuli compared to INs. There was a significant main effect of stimuli type (F (1, 3) = 273.7), P=0.0005, and a significant interaction between stimuli and neuron type (F (1, 3) = 1, 3), P=0.0392 (2-way RM ANOVA). *Q <0.05. N=4 mice. Mean±SEM. **f**, Percent of spinal projection neurons within this study (Warwick) as well as others which reported exact number of responses or percentages to the indicated stimuli. Data replotted from Light [2], Allard [3], Han [4], Bester [5], Keller [6], and Chisholm [7]. **g**, Breakdown of mechanical sensitivity by cardinal modality sensitivity. **h**,**i** Linear regression of preference score vs depth with mean±95% C.I., *P <0.05 (LT, P=<0.0001; Cold, P=<0.0001; Heat, P=0.0046.

Uncorrected *P*-values were calculated via F-test). Consistent with prior literature, thermal stimuli have relatively stronger responses in the superficial layers compared with deeper populations, whereas among the mechanical sub-modalities only the LT stimulus shows any significant correlation to depth. **j**, Cardinal tuning angle as a function of depth. **k**, Cardinal tuning angle and vector amplitude plotted across Lam I (black), Lam IIo (grey) and Lam IIi (white) from most to least dorsal. **l**, Mechanical sub-modality tuning angle across depth. **m**, Mechanical tuning angle and amplitude plotted across Lam I (black), Lam IIo (grey) and Lam IIi (white) from most to least dorsal.

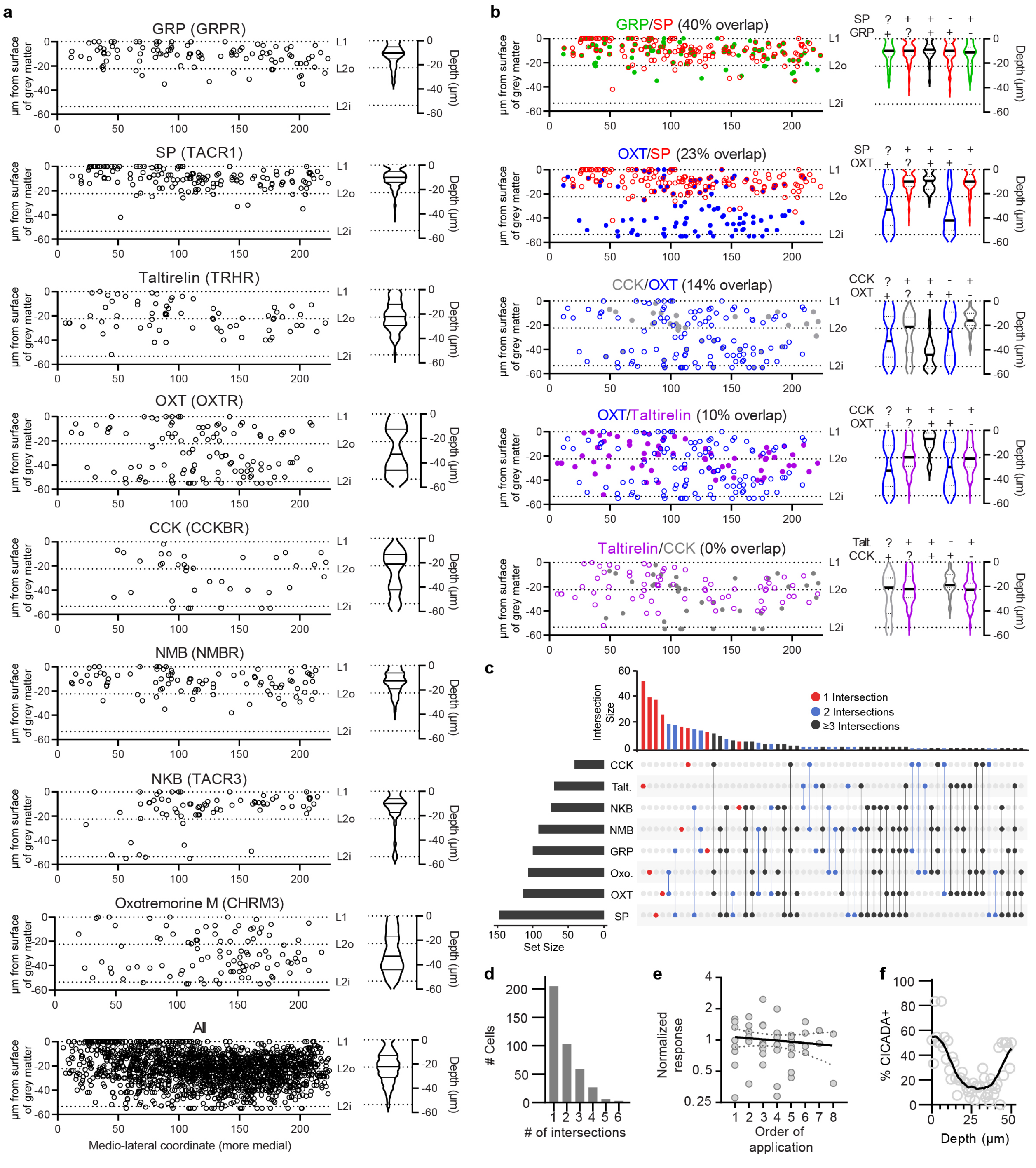

Supplementary Fig. 5 | Characterization of CICADA Ligands

a, Dorso-ventral and medio-lateral distribution of CICADA ligand responses. Individual cells are shown on the left and violin plot of the dorso-ventral distribution on the right. **b**, Assessment of overlap between CICADA ligand responders, individual cells on left and violin plots showing the distribution of the indicated population. For violin plot, '+' indicates a required ligand response, '-' indicates no response and '?' indicates either response is included. **c**, UpSetR plots for all CICADA ligands quantifying the degree of overlap/intersection between populations, 1 response

(red), 2 (blue), and ≥3 (black). **d**, Histogram of ligand co-responsiveness (intersections) from the UpSetR plot in **c**. **e**, Linear regression analysis of normalized ligand response against order of application. No statistically significant effect of application order was found (DFn, DFd (1,45), $Y = -0.02656*X + 1.096$, P=0.42, P value is calculated from an F Test). Data taken from 8 animals with a total of 47 randomized applications. **f**, Percent of cells which responded to 1 or more of the CICADA ligands by depth. The average of individual 1 μm bins are shown with open circles and the smoothed average is shown with a black line.

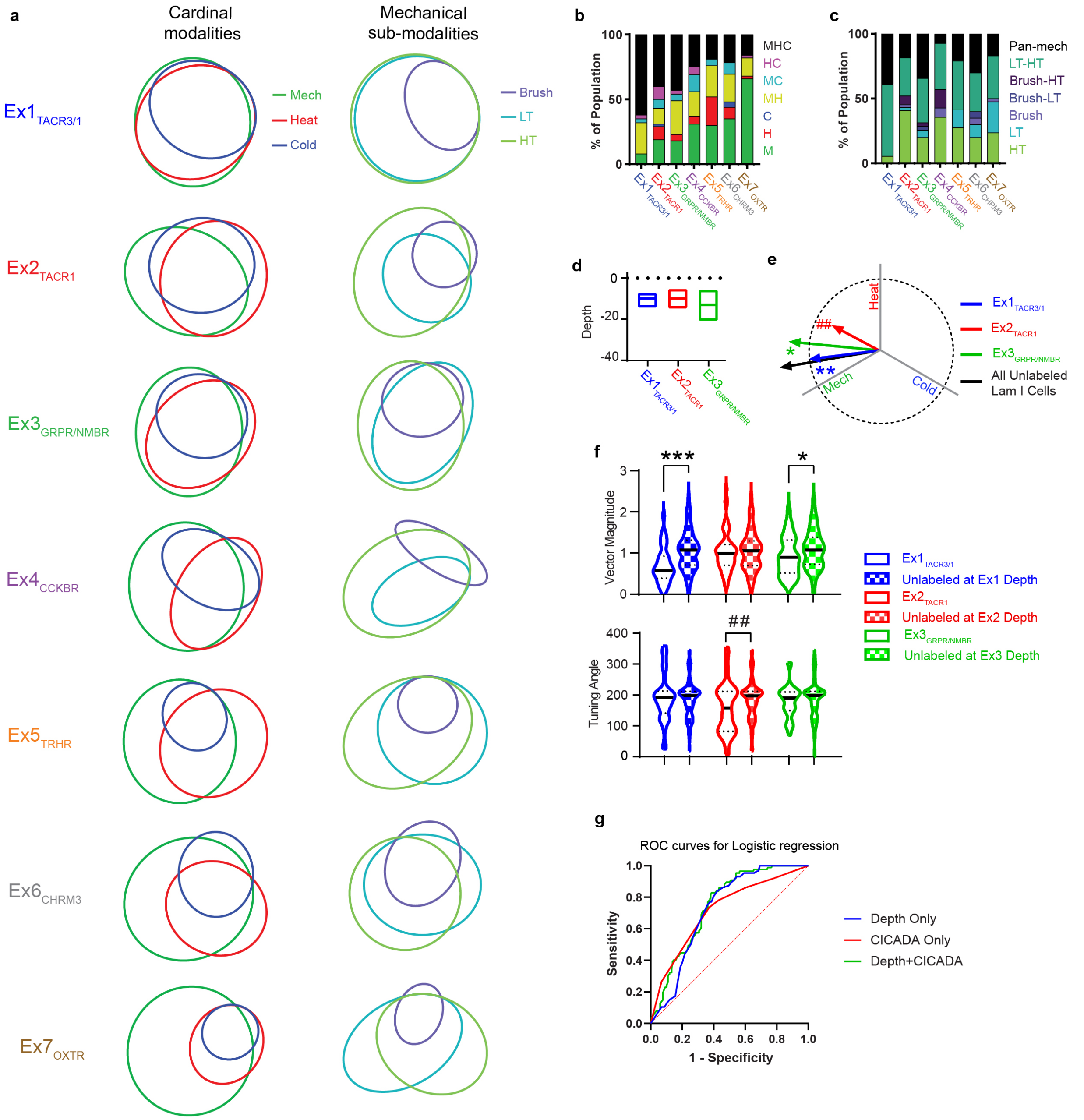

Supplementary Fig. 6 | Extended Characterization of CICADA Populations

a, Proportion of each CICADA population that was responsive to cardinal modalities (cold, mechanical, heat; left) and mechanical sub-modalities (Brush, LT (low-threshold, 0.16 g VF), or HT (high-threshold, 2.0 g VF); Right) shown as a Euler diagram. **b**, Percent of each CICADA population categorized by their cardinal modality: M (Mechanical), H (Heat), or C (Cold) responsive. **c**, Percent of each CICADA population categorized by their mechanical sub-modalities. **d**, Interquartile range (center line, median; box, 25th and 75th percentile) of the depth distribution of lamina I CICADA populations. **e**, Cardinal tuning of lamina I CICADA populations compared to lamina I cells not labeled by CICADA ligands. Vector angle (##*Q*<0.01) and magnitude (**Q*<.05, ***Q*<.001) compared to the average of all lamina I cells. **f**, Cardinal tuning vector magnitude (top) and angle (bottom) for lamina I CICADA populations and excitatory

neurons not labeled by CICADA ligands found at the matching depth. For vector angles: 1-way ANOVA (F (5, 659) = 3.873), P= 0.0282. For vector magnitudes: 1-way ANOVA (F (5, 659) = 4.839), P= 0.0002. Post hoc testing was performed comparing each population's vector angle (##Q<0.01) and magnitude (*Q<.05, ***Q<.001) to the average of the appropriately depth matched cells which were not labeled by CICADA ligands. **g**, Receiver operating characteristic (ROC) curves of 3 logistic regressions predicting Cardinal (MHC) polymodality. The 3 models are dorso-ventral location (depth relative to the dorsal surface), CICADA population, or a multiple logistic regression which includes both variables and interactions. Both depth and CICADA variables were statistically predictive of cardinal polymodality. AUC for each model was compared to a null hypothesis that AUC=0.5, *i.e.* the model is no better than chance at classifying the data. (depth, P<0.001, |Z|=4.34; CICADA cluster, P<0.001, |Z|=3.5). We also found there was a significant interaction between depth and CICADA population (P=0.004, |Z|=2.85) which suggests neither variable can entirely predict the polymodality. For panels **a**-**g**, $Ex1_{TACR3/1}$ = 57 cells, $Ex2_{TACR1}$ = 75 cells, $Ex3_{GRPR/NMBR}$ = 57 cells, $Ex4_{CCKBR}$ = 21 cells, $Ex5_{TRHR}$ = 65 cells, $Ex6_{CHRM3}$ = 49 cells, and $Ex7_{OXTR}$ = 74 cells pooled from 4 mice

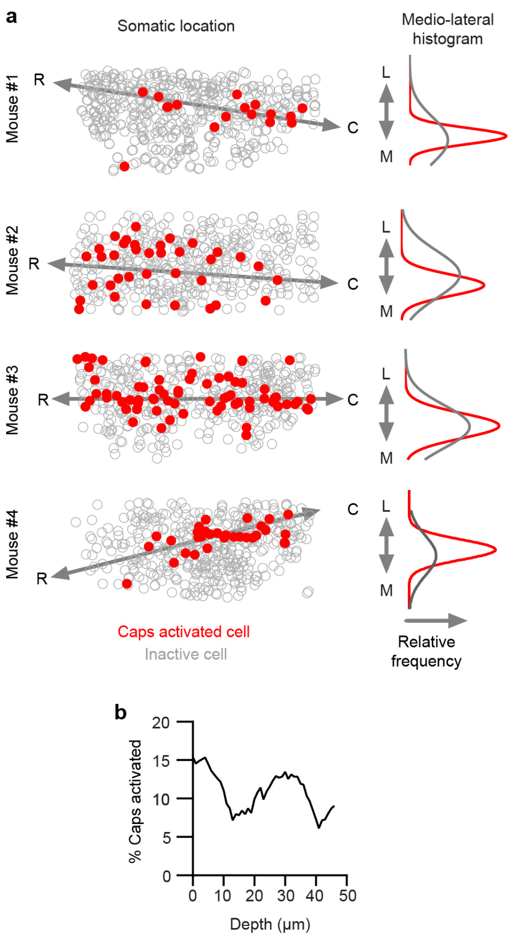

Supplementary Fig. 7 | Primary Responders by mediolateral and dorso-ventral distribution

a, Left: Somatic location in the SDH of neurons which were either quiescent (grey) or activated (red) after capsaicin injection. Right: The relative frequency of capsaicin activated (red) or all cells (grey). Rostral (R), caudal (C), medial (M), and lateral (L) are indicated by the grey arrows. **b**, Percent of cells that were classified as being activated by capsaicin injection (>150% of pre-capsaicin levels based on a 2-minute average of ΔF/F values) across depth.

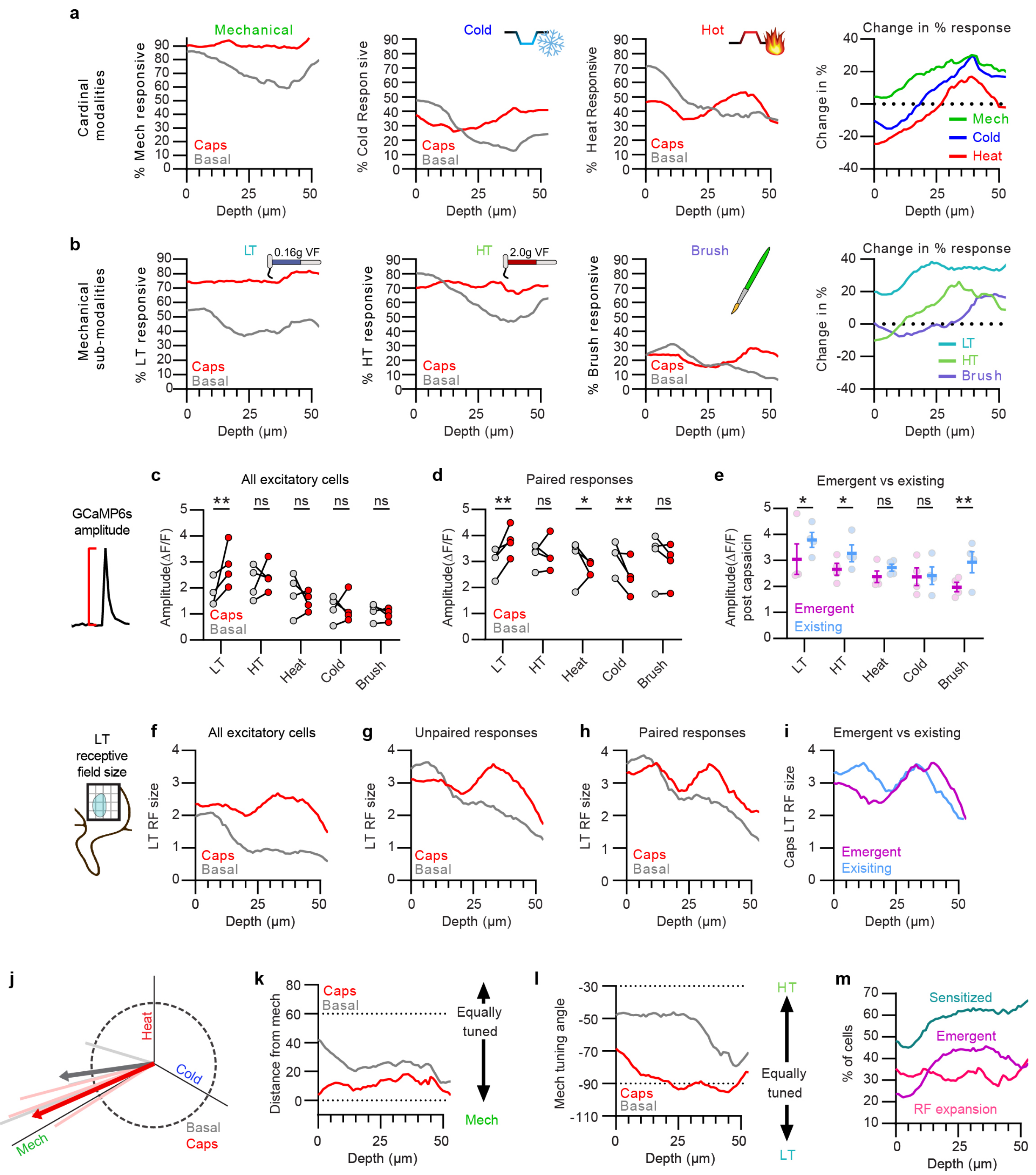

Supplementary Fig. 8 | Alterations in SDH responsiveness after capsaicin vary by depth and basal response

a,**b**, Percent of cells responsive to cardinal (**a**) or mechanical sub-modalities (**b**) before (grey) and after capsaicin (red) across depth. Change in % response on right. **c**, Amplitude among all excitatory cells before/after capsaicin. There was a significant main effect of stimuli type (*F* (4, 12) = 58.14), *P*<0.0001, and a significant interaction between stimuli and Caps (*F* (4, 12) = 6.522), *P*=0.005 (2-way RM ANOVA). **d**, Amplitude among cells which responded to the indicated stimulus both before and after capsaicin (paired responses). There was a significant

main effect of stimuli type (*F* (4, 12) = 5.003), *P*=0.0132, and a significant interaction between stimuli and Caps (*F* (4, 12) = 11.06), *P*=0.0005 (2-way RM ANOVA). **e**, Amplitude after capsaicin among cells which were basally sensitive (blue) versus those which had an emergent response post capsaicin (purple). There was a significant main effect of stimuli type (*F* (4, 12) = 9.017), *P*=0.013 (2-way RM ANOVA). N=4 mice. **f-h**, LT receptive field sizes before/after capsaicin within: all excitatory neurons (**f**), only LT responsive (**g**), only paired LT responses *i.e.* a response before and after capsaicin (**h**). **i**, LT receptive field size post capsaicin in emergent vs existing populations. **j**, Cardin`al tuning of all excitatory neurons. **k**, Cardinal tuning across depth before/after capsaicin. **l**, Mechanical sub-modality tuning across depth before/after capsaicin. **m**, Summary of LT changes post capsaicin. Sensitized cells include cells which had at least one of the following: an emergent response, a receptive field expansion >0.5, or those with a >1.5-fold increase in amplitude. For all tests, **Q* <0.05. N=4 mice, for **c-e** each dot represents the average of all cells within that mouse before or after capsaicin (N=4 mice). All other graphs represent the pooled data from 4 mice with a total of 1,265 cells.

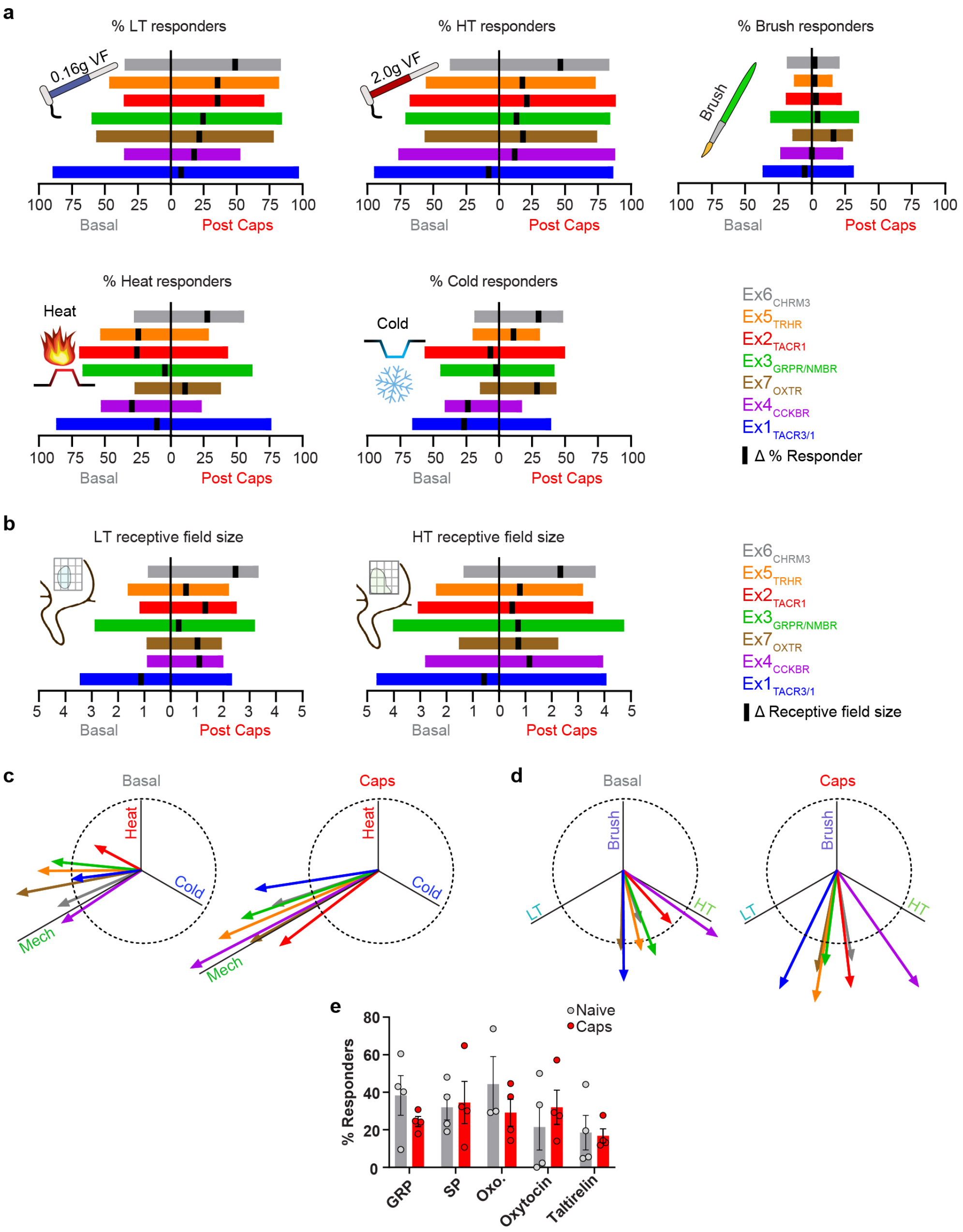

Supplementary Fig. 9 | CICADA Populations show distinct basal response properties and alterations after capsaicin

a, Percent of cells responsive to the indicated modality before (left of y-axis) and after capsaicin treatment (right of y-axis) with the change in % responders (Δ) indicated with a black tick on the right axis for an increase in the percent of cells responsive and on the left axis for a decrease. CICADA populations are color coded and arranged in order of high to low change in low-threshold (LT) percent responders. Bars are the mean value of 4 animals. **b**, Receptive field sizes for low-threshold and high-threshold (HT) stimulations before/after capsaicin with the change (Δ) in RF size indicated by a black tick mark where increases in RF size are on the right and decreases on the left. CICADA populations are arranged as described in **a**. Bars are the mean value of 4 animals. **c**, Cardinal tuning of Ex1-7 before (left) and after capsaicin (right). **d**,

Mechanical sub-modality tuning of Ex1-7 before (left) and after capsaicin (right). Tuning vectors are calculated as the mean of all pooled cells in each cluster. $Ex1_{TACR3/1}$ = 57 cells, $Ex2_{TACR1}$ = 75 cells, $Ex3_{GRPR/NMBR}$ = 57 cells, $Ex4_{CCKBR}$ = 21 cells, $Ex5_{TRHR}$ = 65 cells, $Ex6_{CHRM3}$ = 49 cells, and $Ex7_{OXTR}$ = 74 cells from 4 mice. **e**, Percent of CICADA labeled cells responsive to the indicated CICADA ligands averaged by animal in either naïve preparations or animals which were subjected to the cutaneous stimulation protocol and capsaicin injection described in Fig. 6b (Caps). No significant differences between naïve and capsaicin-treated preparations were found.

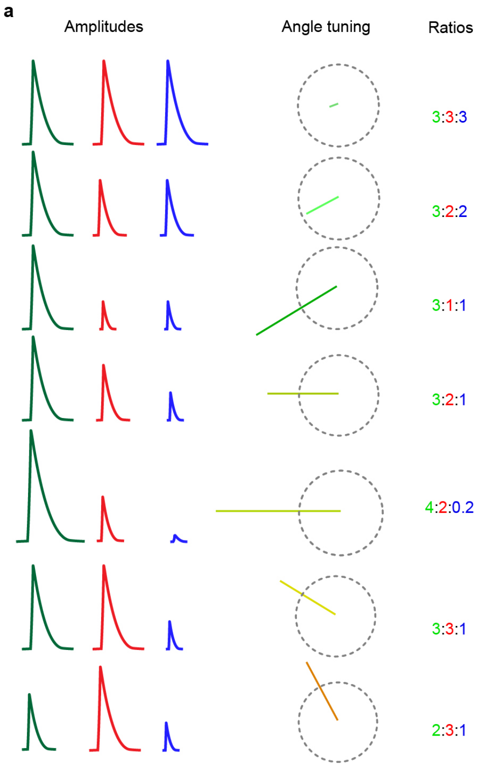

Supplementary Fig. 10 | Interaction between amplitude of response and vector tuning angle and magnitude

a, Examples of amplitudes of response (left), the ratios of response (right) and the resultant vector tuning for cardinal modalities (mechanical, green; red, heat; blue, cold). Multiple examples are given in order to exemplify how the angle and magnitude represent different tunings. *e.g*. the first 3 examples all have the same mechanical (green) response but as the relative ratios of mechanical to heat/cold are increased, the magnitude of the tuning is increased with maintaining the same angle indicating a stronger preference for mechanical relative to the other modalities permitting an assessment of preference within a polymodal cell.

Cluster	Ex1 (TACR3/1)	Ex2 (TACR1)	Ex3 (GRPR/NMBR)	Ex4 (CCKBR)	Ex5 (TRHR)	Ex6 (CHRM3)
Ex2 (TACR1)	0.3221					
Ex3 (GRPR/NMBR)	0.1015	0.1015				
Ex4 (CCKBR)	0.0192	0.0192	0.0879			
Ex5 (TRHR)	0.0001	0.0001	0.0001	0.0495		
Ex6 (CHRM3)	0.0001	0.0001	0.0001	0.0001	0.0001	
Ex7 (OXTR)	0.0001	0.0001	0.0001	0.0001	0.0001	0.0053

Supplementary Table 1 | Full statistical testing results for CICADA population depths.

A 1-way ANOVA was performed for the depth of CICADA populations. There was a significant difference among the means (F (6, 390) = 9.312), P=<0.0001. Post hoc testing was performed comparing each population mean depth to each other population and the false-discovery rate corrected Q-value is reported for each comparison with values Q<0.05 shaded indicating a discovery. P-values of less than 0.0001 are reported by Prism as <0.0001 rather than exact values.

Author	PMID	Species	Method	Anesthesia or Ex vivo	% Brush or Light Touch	Brush Pressure	% Pinch	Pinch Pressure	Mech	Heat	Cold	MHC	Any poly-modality	Thermal Method
Han[4]	10195146	Cat	Intracellular Recording	Pentobarbital	**Data not shown**	Not quantified	**66%**	Not quantified	**66%**	**45%**	**52%**	**18%**	**45%**	Water jet
Light[2]	8237218	Cat	Intracellular Recording	Pentobarbital	**9%**	Not quantified	**79%**	Not quantified	**88%**	**23%**	**23%**	**0%**	**34%**	Thermode
Warwick	Current Work	Mouse	Multiphoton Ca2+ Imaging	Ex vivo	**53%**	6.5 kPa	**--**	Not Applied	**100%**	**52%**	**47%**	**48%**	**52%**	Thermode
Hachisuka[8,9]	27991851 + 31577643	Mouse	Patch Clamp Recording	Ex vivo	**Data not shown**	Not quantified	**--**	Not Applied	**70%**	**33%**	**59%**	**43%**	**41%**	Water jet
Allard[3]	30719699	Mouse	Extracellular Recording	Isoflurane	**23.21%**	Not quantified	**100%**	2370 mN (no area)	**83%**	**83%**	**64%**	**~50%***	**80%**	Water jet
Chisholm[7]	33769365	Mouse	Epiflourescence Ca2+ Imaging	Urethane	**0%**	Not quantified	**42%**	Not quantified	**72%**	**25%**	**85%**	**18%**	**70%**	Thermode
Bester[5]	10758132	Rat	Extracellular Recording	Halothane & nitrous oxide	**25%**	Not quantified	**92%**	Not quantified	**92%**	**100%**	**32%**	**35%**	**92%**	Water jet
Keller[6]	17900333	Rat	Extracellular Recording	Pentobarbital or ketamine/xylazine	**21%**	Not quantified	**100%**	1250 kPa (100g/1mm)	**100%**	**N/A**	**N/A**	**N/A**	**N/A**	N/A

Supplementary Table 2 | Comparison of spinal projection neuron studies

Data from studies which recorded from spinal projection neurons (encompassing spino-thalamic and spino-parabrachial) and reported data which could be usefully compared, *e.g.* studies which reported exact numbers of responses to brush or pinch out of a total were included but studies which qualitatively described typical results or did not show exact quantification were not included. Brush and light touch were presented as equivalent stimuli in a number of studies and so this column included either if the paper showed them separately. To collate the % of neurons responsive to Mechanical (Mech), Heat, and Cold stimuli any stimulus of that cardinal modality was considered a response, *i.e.* if multiple types of mechanical stimuli were presented (e.g. brush and pinch) a response to any of made that cell mechanically responsive. *This is an estimate of the MHC polymodality within Allard *et al* based-on counting data points in their graphs. While the number of MH cells was explicitly mentioned, the number of cells which also responded to cooling as well as MH was not, and we counted any cell on their summary graph with >1 AP to cooling as a response. No attempt was made to compare subtypes such as nociceptive specific neurons across studies.

Supplementary References

1 Wang, F. *et al.* Sensory Afferents Use Different Coding Strategies for Heat and Cold. *Cell Rep* **23**, 2001-2013, doi:10.1016/j.celrep.2018.04.065 (2018).

2 Light, A. R., Sedivec, M. J., Casale, E. J. & Jones, S. L. Physiological and morphological characteristics of spinal neurons projecting to the parabrachial region of the cat. *Somatosens Mot Res* **10**, 309-325, doi:10.3109/08990229309028840 (1993).

3 Allard, J. Physiological properties of the lamina I spinoparabrachial neurons in the mouse. *J Physiol* **597**, 2097-2113, doi:10.1113/JP277447 (2019).

4 Han, Z. S., Zhang, E. T. & Craig, A. D. Nociceptive and thermoreceptive lamina I neurons are anatomically distinct. *Nat Neurosci* **1**, 218-225, doi:10.1038/665 (1998).

5 Bester, H., Chapman, V., Besson, J. M. & Bernard, J. F. Physiological properties of the lamina I spinoparabrachial neurons in the rat. *J Neurophysiol* **83**, 2239-2259, doi:10.1152/jn.2000.83.4.2239 (2000).

6 Keller, A. F., Beggs, S., Salter, M. W. & De Koninck, Y. Transformation of the output of spinal lamina I neurons after nerve injury and microglia stimulation underlying neuropathic pain. *Mol Pain* **3**, 27, doi:10.1186/1744-8069-3-27 (2007).

7 Chisholm, K. I. *et al.* Encoding of cutaneous stimuli by lamina I projection neurons. *Pain* **162**, 2405-2417, doi:10.1097/j.pain.0000000000002226 (2021).

8 Hachisuka, J. *et al.* Semi-intact ex vivo approach to investigate spinal somatosensory circuits. *Elife* **5**, doi:10.7554/eLife.22866 (2016).

9 Hachisuka, J., Koerber, H. R. & Ross, S. E. Selective-cold output through a distinct subset of lamina I spinoparabrachial neurons. *Pain* **161**, 185-194, doi:10.1097/j.pain.0000000000001710 (2020).
