# Supplementary material for: Cell type-specific calcium imaging of central sensitization in mouse dorsal horn
Source: Nat Commun. 2022 Sep 3;13:5199. doi: 10.1038/s41467-022-32608-2 (PMC9440908; doi:10.1038/s41467-022-32608-2)
Supplement: Supplementary file 3 — Reporting Summary [file 41467_2022_32608_MOESM3_ESM.pdf]

nature portfolio

Corresponding author(s): H. Richard Koerber and Sarah E. Ross

Last updated by author(s): Jul 28, 2022

Reporting Summary

Nature Portfolio wishes to improve the reproducibility of the work that we publish. This form provides structure for consistency and transparency in reporting. For further information on Nature Portfolio policies, see our Editorial Policies and the Editorial Policy Checklist.

Statistics

For all statistical analyses, confirm that the following items are present in the figure legend, table legend, main text, or Methods section.

n/a	Confirmed	
☐	☒	The exact sample size (n) for each experimental group/condition, given as a discrete number and unit of measurement
☐	☒	A statement on whether measurements were taken from distinct samples or whether the same sample was measured repeatedly
☐	☒	The statistical test(s) used AND whether they are one- or two-sided<br>*Only common tests should be described solely by name; describe more complex techniques in the Methods section.*
☒	☐	A description of all covariates tested
☐	☒	A description of any assumptions or corrections, such as tests of normality and adjustment for multiple comparisons
☐	☒	A full description of the statistical parameters including central tendency (e.g. means) or other basic estimates (e.g. regression coefficient) AND variation (e.g. standard deviation) or associated estimates of uncertainty (e.g. confidence intervals)
☐	☒	For null hypothesis testing, the test statistic (e.g. F, t, r) with confidence intervals, effect sizes, degrees of freedom and P value noted<br>*Give P values as exact values whenever suitable.*
☒	☐	For Bayesian analysis, information on the choice of priors and Markov chain Monte Carlo settings
☒	☐	For hierarchical and complex designs, identification of the appropriate level for tests and full reporting of outcomes
☒	☐	Estimates of effect sizes (e.g. Cohen's d, Pearson's r), indicating how they were calculated

Our web collection on statistics for biologists contains articles on many of the points above.

Software and code

Policy information about availability of computer code

Data collection	Calcium imaging data collection was performed using commercial Leica LAS AF imaging software (Version 2.7.3.9723). Peltier temperature was collected with the commercial Signal 7 (CED). Pressure measurements were collected through the open-source Arduino Integrated Development Environment using an Arduino library for load scales located here: https://github.com/bodge/HX711.
Data analysis	Image files were viewed and organized using the open-source program FIJI (V1.53i, ImageJ2) to prepare the files for image stabilization. Image stabilization was performed in the open source Suite2p (v0.10.0, HHMI Janelia) using both rigid and non-rigid motion correction modules. The stabilized image files were inspected for movement artifacts and ROIs analysis in FIJI. Data traces were then normalized and quantified in the commercial software Excel (Microsoft, Version 2206) and then the results were analyzed Prism (V9.1, GraphPad). Euler diagrams were made using the open source eulerAPE (V3, Luana Micallef and Peter Rodgers). Vectors were made in the commercial software Matlab (R2021a, Mathworks). K-Means clustering and t-SNE visualization was performed in the open-source package Orange (v3.26, University of Ljubljana, Slovenia). G* Power (University of Düsseldorf, Germany, Version 3.1.9.4) was used to perform power analyses. All code generated by this work for image processing and analysis are located at: https://github.com/cawarwick

For manuscripts utilizing custom algorithms or software that are central to the research but not yet described in published literature, software must be made available to editors and reviewers. We strongly encourage code deposition in a community repository (e.g. GitHub). See the Nature Portfolio guidelines for submitting code & software for further information.

Data

Policy information about availability of data

All manuscripts must include a data availability statement. This statement should provide the following information, where applicable:

- Accession codes, unique identifiers, or web links for publicly available datasets
- A description of any restrictions on data availability
- For clinical datasets or third party data, please ensure that the statement adheres to our policy

All data analyzed for this study are included in the article, supplementary figures, tables, and source data. The raw imaging files are available at the following GIN (G-Node Infrastructure) repository: https://gin.g-node.org/warwickc/CICADAv1Caps. Parabrachial nucleus injections were verified by utilizing the annotated mouse anatomical database in the publicly available Allen Mouse Brain Reference Atlas (https://mouse.brain-map.org/static/atlas). Comparisons to Russ et al (1) were made using the publicly available harmonized atlas of mouse spinal cord cell types (https://seqseek.ninds.nih.gov/).

Field-specific reporting

Please select the one below that is the best fit for your research. If you are not sure, read the appropriate sections before making your selection.

[x] Life sciences   [ ] Behavioural & social sciences   [ ] Ecological, evolutionary & environmental sciences

For a reference copy of the document with all sections, see nature.com/documents/nr-reporting-summary-flat.pdf

Life sciences study design

All studies must disclose on these points even when the disclosure is negative.

Sample size	We estimated our sample size with G*Power. Our original pilot data suggested that ~40% of cells were sensitized to LT stimulation after capsaicin treatment. Using these as probabilities, assuming an alpha of 0.05, power of 80%, and a strong association for a CICADA defined neuron covariate (R2=0.49), we find that approximately 130 neurons would need to be sampled. Correcting for the originally estimated 10 CICADA populations (some population were merged during K-means clustering), we infer that a total of 1300 neurons would need to be sampled. Based on our preliminary data, we found each imaging session to have an average of 300 neurons; thus, ~4 animals were needed.
Data exclusions	Any cells that showed significant deviation from their XY location, signs of Z-drift (e.g., changes in the appearance of the nucleus, soma, or processes), blebbing, or cell death in the latter portions of the recording were excluded from analysis. Once the masks were finalized, the mean fluorescence was calculated and any cell that has significant drift (>200%) in their 5-min binned median fluorescence value was excluded because such a change was indicative of either physical drift or Ca2+ dysregulation which would signal signs of apoptosis. Theses metrics are standard for our lab when considering long term recordings of cells ex vivo.
Replication	We performed 4 replicates of the capsaicin injection experiment using 4 different animals each from a different litter with 2 male and 2 female animals all of which showed consistently showed increased sensitivity to low-threshold stimulation after injection. Our study was not powered to detect sex differences and a low powered analysis did not show any significant trends so both sexes were merged. We used 8 animals with a total of 47 randomized applications for testing the stability of CICADA to consistently identify the same neuronal populations.
Randomization	The presentation order of natural stimuli was randomized within each testing block (i.e. baseline or post capsaicin testing periods). CICADA agonist application was also randomized. A repeated measures experimental design was utilized, so we compared each animal to its baseline period and each animal's baseline period was used was to control for covariates, e.g. age, sex, amount of peripheral skin input, health of preparation, et cetera.
Blinding	The investigator performing the imaging was not blinded to the application of the stimuli as it would be impossible to blind the investigator applying the stimulus to the skin. Where possible, we used electronically controlled rather than manually applied stimuli to limit these problems. For analysis, the investigators were not blinded to baseline or capsaicin as the effects of capsaicin stimulation were obvious to the naked eye looking at the recordings.

Reporting for specific materials, systems and methods

We require information from authors about some types of materials, experimental systems and methods used in many studies. Here, indicate whether each material, system or method listed is relevant to your study. If you are not sure if a list item applies to your research, read the appropriate section before selecting a response.

Materials & experimental systems

n/a	Involved in the study
☒	☐ Antibodies
☒	☐ Eukaryotic cell lines
☒	☐ Palaeontology and archaeology
☐	☒ Animals and other organisms
☒	☐ Human research participants
☒	☐ Clinical data
☒	☐ Dual use research of concern

Methods

n/a	Involved in the study
☒	☐ ChIP-seq
☒	☐ Flow cytometry
☒	☐ MRI-based neuroimaging

Animals and other organisms

Policy information about studies involving animals; ARRIVE guidelines recommended for reporting animal research

Laboratory animals	All experiments were performed with approval of the University of Pittsburgh's Institutional Animal Care and Use Committee (IACUC, Protocol Numbers 21100045 and 21038819). Mice, from a C57/BL6 background, that were heterozygous for both the Vglut2-ires-cre allele (Jax Stock #016963) the Ai96 allele (for Cre-dependent expression of GCaMP6s, Jax Stock # 028866) were used for all experiments. Mice were housed on a 12-hour light cycle with ad-libitum water and food in accordance with the United States National Institutes of Health guidelines for the care and use of laboratory animals. Relative humidity was kept between 30% and 70% and temperature was kept between 20 and 26°C. 10-15 fresh air changes per hour was provided for adequate ventilation. Daily observation of all animals was required, and bedding was changed at least weekly depending on the number of animals. Social housing was utilized with no more than 4 adult mice per cage was permitted. Single housing was not used except for cases of animal welfare, e.g., aggressive males being separated. Enrichment was provided in the form of soft bedding that can be burrowed within, plastic housing domes, and running wheels. Ca2+ imaging was performed on mice of both sexes ranging from 5-10 weeks of age ranging from 20-25 grams depending on sex and age.
Wild animals	No wild animals were used in this study.
Field-collected samples	No field collected samples were used in this study.
Ethics oversight	All experiments were performed with approval of the University of Pittsburgh's IACUC.

Note that full information on the approval of the study protocol must also be provided in the manuscript.
